# WoundPy: an improved automatized method for in vitro cell motility investigation based on wound alignment and absolute velocity profiling

**DOI:** 10.3389/fcell.2026.1919916

**Published:** 2026-09-07

**Authors:** E. Galante, M. Bizzarri, N. Monti, G. Lentini

**Affiliations:** 1 Department BeSSA - Wellbeing, Health and Environmental Sustainability, Sapienza University of Rome, Rome, Italy; 2 System Biology Group Lab, Rome, Italy; 3 Bioluna Srl, Rome, Italy

**Keywords:** migration, optical microscopy, python, software, wound healing assay

## Abstract

The Wound Healing Assay is a standard technique for studying cell motility, yet it faces challenges in reproducibility and data interpretation. Here we present WoundPy, a Python-based executable software featuring a user-friendly interface for semi-automatic, researcher-supervised Region of Interest detection. WoundPy streamlines image analysis and management of replicates, providing rapid graphical outputs. A key pre-processing feature is the automated vertical wound alignment, which eliminates operator-dependent errors typical of optical microscopy. Velocity results are calculated as absolute values from multi-time-point imaging. The software was tested on three datasets from biological experiments, and a comparison with ImageJ Wound Healing Size tool revealed a significant underestimation of the wound area by the latter compared to WoundPy. In conclusion, this new software offers an extremely streamlined approach that easily enables to perform an analysis that is both accurate and fast, drastically reducing the time required for both numerical data acquisition and its subsequent analysis.

## Introduction

Notwithstanding advances in cell culture techniques and analytical methodologies, the wound healing assay remains necessary in many cases to conduct experiments using two-dimensional culture models for investigating cell motility. Wound healing is a complex cellular and biochemical process necessary to restore structurally damaged tissue (Grada et al., 2017). This intricate mechanism encompasses phenomena such as cytoskeletal remodelling (Pollard and Borisy, 2003), the acquisition of cell polarity associated with the redistribution of membrane phospholipids (Lentini et al., 2025) and adhesion molecules (Burridge and Chrzanowska-Wodnicka, 1996), as well as the formation of Lamellipodia, Filopodia, Pseudopodia, or Invadopodia - cytosolic protrusions that drive and determine the cell movement (Friedl and Wolf, 2003).

Cell migration guides morphogenic processes during embryogenesis and remains pivotal in the adult organism for physiological and pathological responses. Notably, it drives leukocyte recruitment during inflammation and is essential for the fibroblast and endothelial cell movements required for wound healing (Lauffenburger and Horwitz, 1996). Mostly, cell motility plays a prominent role in the study of pathological conditions. For instance, a cancer cell undergoing epithelial-mesenchymal transition acquire the ability to activate migratory and invasive program. These processes allow neoplastic cells to enter lymphatic and blood vessels for dissemination into the circulation, and then undergo metastatic growth in distant organs (Friedl and Wolf, 2003). Most notably in cancer-related settings, the use of techniques such as the Wound Healing Assay (WHA) facilitates the assessment of the capability of the molecules under investigation to reduce the invasive and migratory potential of cancer cells. This allows an efficient preliminary screening strategy (Hulkower and Herber, 2011), enabling the early exclusion of compounds that are ineffective in reducing the motility of metastatic cells *in vitro*, thereby preventing the unnecessary and economically demanding application of downstream molecular analyses.

However, it is well known in the literature that the different analysis software tools currently in use are labour intensive, inaccurate (Vang Mouritzen and Jenssen, 2018) and the scratch wound assay has low reproducibility (Guy et al., 2017). This is mainly attributable to operator errors during wound alignment lacking live imaging support, as well as the automated Region of Interest (ROI) detection found in some tools. Such automation not only restricts user intervention but also frequently introduces algorithm-dependent systematic errors. Therefore, although WHA, as it is currently known, may be regarded as an outdated technique, it remains a cornerstone of basic research since, when properly analyzed, it enables the rapid and reproducible acquisition of important data.

To date, existing free tools for WHA analysis are primarily designed to minimize operator error during the detection of the wound area (ROI) (De Cillis et al., 2025; Doğru et al., 2024; Erdem et al., 2021; Yaothak et al., 2025). Notably, the ImageJ Wound Healing Size Tool (Suarez-Arnedo et al., 2020) employs a macro based on variance calculation, which exploits the texture difference between the high-variance cell monolayer and the smooth, cell-free gap to segment the ROI via intensity thresholding. While this tool allows the researcher to adjust critical parameters - including the variance filter radius, saturation percentage, and threshold values - it lacks real-time interactivity, requiring the entire analysis pipeline to be executed *de novo* for every parameter modification. This workflow significantly hinders the throughput of WHA analysis, particularly for large-scale experiments involving multiple experimental conditions, time points, and numerous replicates. Moreover, the ImageJ tool often fails to accurately detect wounds that are near closure or exhibit only small open areas. More advanced computational tools use complex image processing algorithms, each optimized for specific advantages: some specialize in refining automatic ROI detection across extensive datasets for long-duration time-lapse series (Ascione et al., 2017a; Garcia-Fossa et al., 2020), while others are tailored for high-resolution motility assays (Doğru et al., 2024), offering precise discrimination between cell-covered regions and cell-free gaps. However, few of these tools facilitate direct comparison between experimental conditions and time points (De Cillis et al., 2025; Gebäck et al., 2009), thereby relegating the post-processing of raw data to the user.

Certain tools enable velocity calculation, variously defined as mean front velocity (Garcia-Fossa et al., 2020; Kauanova et al., 2021; Jonkman et al., 2014; Bahar and Yoon, 2021), closure rate (Gnerucci et al., 2020; Ascione et al., 2017b), or local velocity (Vašinková et al., 2025; Scheda et al., 2021; C et al., 2018; Varankar and Bapat, 2018). Mean front velocity is typically derived as half the rate of wound width reduction over time; however, without effective automation in determining the mean front position, this approach is prone to significant operator error. Conversely, closure rate is calculated as the time derivative of the percentage change in open area, providing only a relative measure rather than an absolute quantification of cell motility. Finally, sophisticated software suites allow for the calculation of absolute local velocity, but often necessitate advanced instrumentation - such as lens-free imaging for high-frequency temporal acquisition or fluorescence labeling for single-cell tracking in live microscopy. Consequently, these approaches are often cost-prohibitive and require technical resources beyond standard light microscopy. Lastly, few tools facilitate direct image-overlay analysis to visualize newly covered regions, a feature generally restricted to automated workflows that ensure a fixed field of view (Garcia-Fossa et al., 2020; Scheda et al., 2021). When images are acquired manually, such analysis is susceptible to significant error arising from human operator misalignment.

To the best of our knowledge, no currently available open-source tool featuring a user-friendly interface - executable without programming expertise - simultaneously provides time-efficient ROI detection for WHA images, scratch area measurement, direct comparison between time points within the same replicate, and aggregated quantitative analysis for comparing different conditions across biological replicates. In essence, despite the ease of image acquisition, the analytical pipeline for WHA remains incompletely automated.

**FIGURE 1:**
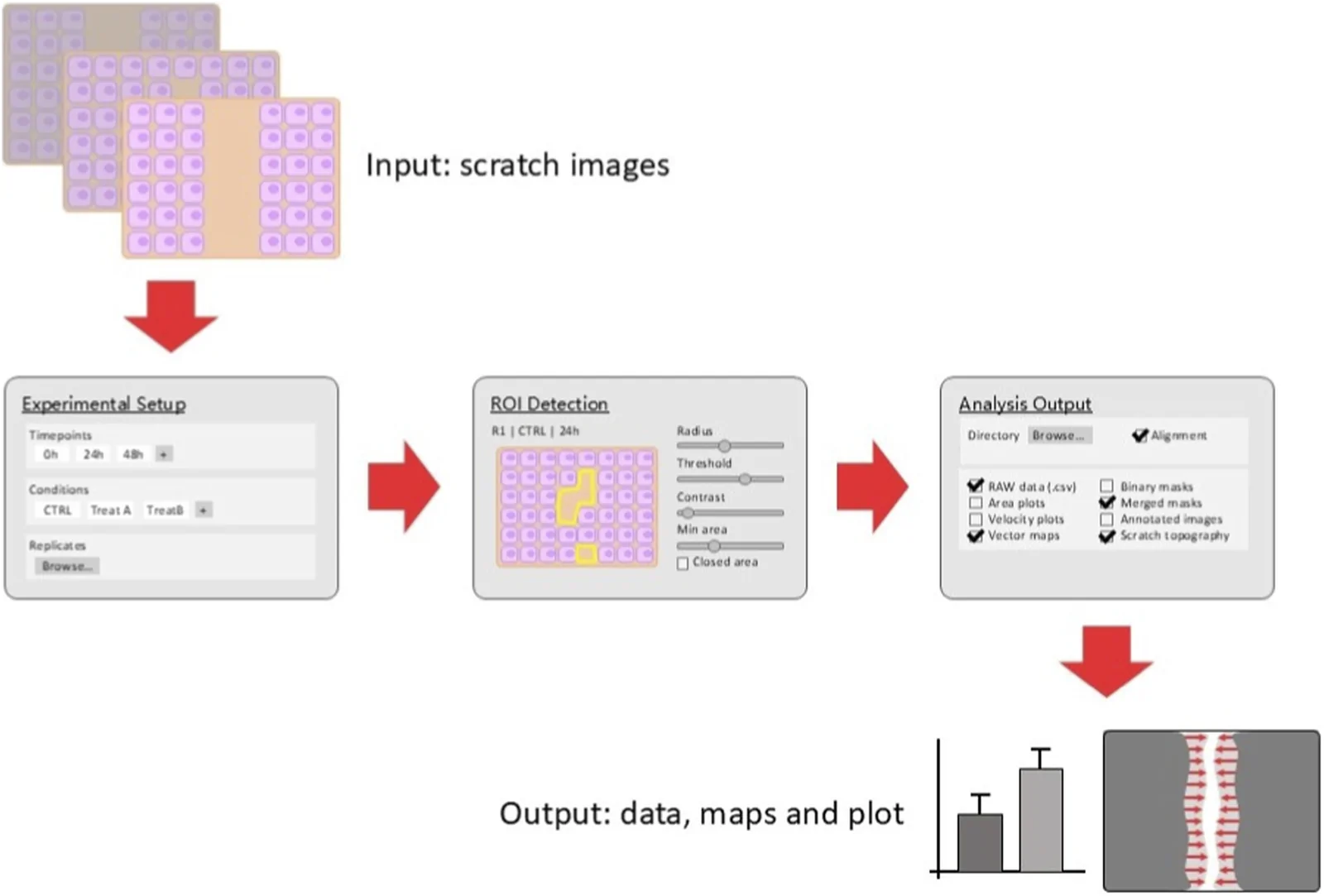
Schematic of WoundPy’s workflow. The tool takes as input all images from all replicates, requires the experimental setup configuration and the dynamic definition of ROIs; finally, it returns the requested outputs. More details on the use of the tool are provided in the Results, Tool structure section.

Here we present WoundPy, a Python-based standalone executable tool (.exe and.app) designed to drastically reduce WHA analysis time. The tool features rapid experimental setup configuration and enables dynamic, user-guided ROI correction via the adjustment of intuitive parameters (threshold, variance radius, contrast, and minimum area). Additionally, WoundPy introduces a wound alignment function across time points, facilitating accurate analysis and cleaner data presentation even for standard light microscopy datasets. This alignment capability further expands visualization options through topological image analysis. Moreover, a key innovative feature of WoundPy is the ability to calculate the absolute velocities along the entire wound front across time points via automated ROI analysis. Following rapid ROI definition, the software yields quantitative data on wound areas and velocities (including means and standard deviations across replicates), time-dependent plots for area and velocity, cumulative histograms, topological analysis plots, and time-point overlay images for effective data presentation. The WoundPy workflow is illustrated in Figure 1.

## Methods

### Image acquisition and datasets

The images used for the validation of WoundPy originate from three distinct datasets. The images from the first two experimental setups were acquired using a MotiCam S6 camera on an AE 2000 (Motic) optical microscope at ×4 magnification, with a scale of 192 pixels/mm. The images from the third setup belong to a public image set BBBC019v2 from the Broad Bioimage Benchmark Collection (Ljosa et al., 2012); specifically we used the MDCK set of images, acquired with an LSM-510 microscope (Zeiss, Germany) in non-confocal mode. The first dataset consist of images acquired for the study by (Monti et al., 2025), while the second dataset was generated within the scope of an ongoing project. The first experimental setup consisted of three biological replicates using the MDA-MB-231 cell line, with three time points (0 h, 24 h, and 30 h) and two conditions (CTRL and SL-treated). The second setup included four replicates using MDA-MB-231 cells, evaluated at two time points (0 h and 24 h) across three conditions (CTRL, TreatA, and TreatB, where treatments represent potential motility inhibitors). The third setup included a single replicate using MDCK/YFP-membrane cells, with two timepoints and several conditions. Since the timepoints and conditions are not declared, timepoints of 0 h and 24 h were assumed for the test, and the conditions were renamed to L1, L2, L4, L5, L6, and L7, following the names of the images available in the dataset. The three experimental setups used for WoundPy validation are summarized in Table 1.

**TABLE 1:** Summary of the experimental setups used to validate WoundPy and compare results with data obtained from other tools.

Experimental setup	Cell line	Timepoints (h)	Conditions	n° of replicas
1	MDA-MB-231	0, 24, 30	CTRL, SL	3
2	MDA-MB-231	0, 24	CTRL, TreatA, TreatB	4
3	MDCK/YFP	0, 24	L1, L2, L4, L5, L6, L7	1

### Software architecture

WoundPy is a Python-based tool (version 3.12.6; Python Software Foundation, https://www.python.org/). To run the tool directly from the source code, users must install the required dependencies using the provided requirements.txt file (which pins the exact library versions used) and execute the main.py script. The software is compiled into a standalone executable (.exe and.app) and is structured around three core open-source Python files: main.py, pages.py, and analyzer.py. The first module handles application initialization and frame navigation, the second manages the Graphical User Interface (GUI) and input parameter collection, while the third contains all computational algorithms for data output. These algorithms are described in the following sections.

#### ROIs detection

The ROI recognition method is implemented in the detect_wound_area function using the Python OpenCV (Gollapudi, 2019) and NumPy (Harris et al., 2020) libraries. First, the parameters radius of variance, threshold, contrast and minimum area are collected. Then image is converted from BGR to grayscale (cv2.cvtColor). Subsequently, *Histogram Stretching* is applied, controlled by the contrast parameter. A saturation percentage is calculated as sat=contrast / 10 . The percentiles mi and ma of the gray distribution are then computed based on sat and 100−sat . The image is clipped and normalized so that pixels between mi and ma span the full 0–255 range. This step enhances the cell texture against the flat background, even under suboptimal lighting conditions.

self.default_params = {“radius”: 20, “threshold”: 80, “contrast”: 1, “min_area”: 200, “is_closed”: False}
  def detect_wound_area(self, img_bgr, params=None):
    if params is None: params = self.default_params
    if params.get(“is_closed”, False):
     return np.zeros((img_bgr.shape[0], img_bgr.shape[1]), dtype=np.uint8)
  gray = cv2.cvtColor(img_bgr, cv2.COLOR_BGR2GRAY)
  contrast = params.get(“contrast”, 1)
  sat = contrast / 10
    if sat > 0:
    mi, ma = np.percentile(gray, (sat, 100 - sat))
    gray = np.clip((gray - mi) * (255.0 / (ma - mi + 1e-5)), 0, 255).astype(np.uint8)

The algorithm exploits the high local variance (complex texture) of the cell monolayer compared to the low variance (uniform color) of the cell-free gap. A kernel of size $k=\left(2*radius\right)+1$ is defined. The local mean ( μ ) and the mean of squares ( $\mu^{2}$ ) are calculated by applying a box filter (cv2.blur) with dimensions k×k . The local variance is computed using the standard computational formula as in Equation 1:

$$Var=E\left[X^{2}\right]-{\left(E\left[X\right]\right)}^{2}$$ (1)

In the code, this is implemented as mu2−mu * mu . The result is normalized (cv2.normalize) to the 0–255 range (8-bit) to obtain a “variance map”. For binarization, a fixed à is applied to the variance map using the threshold parameter. Since the wound exhibits low variance, an inverse threshold (THRESH_BINARY_INV) is used: pixels with variance below the threshold value become white (255), while others become black (0). To remove noise (small holes in the monolayer or islands within the wound), morphological operations with a 5×5 elliptical structuring element are applied (opening removes small background noise; closing fills small black holes within the white mask).

k = (params.get(“radius”, 20) * 2) + 1
f = gray.astype(np.float32)
mu = cv2.blur(f, (k, k))
mu2 = cv2.blur(f * f, (k, k))
var = cv2.normalize(mu2 - mu * mu, None, 0, 255, cv2.NORM_MINMAX).astype(np.uint8)_, mask = cv2.threshold(var, params.get(“threshold”, 100), 255, cv2.THRESH_BINARY_INV)
kern = cv2.getStructuringElement(cv2.MORPH_ELLIPSE, (5, 5))
mask = cv2.morphologyEx(mask, cv2.MORPH_OPEN, kern, iterations=2)
mask = cv2.morphologyEx(mask, cv2.MORPH_CLOSE, kern, iterations=2)

Finally, contours are identified (cv2.findContours). A minimum admissible area is calculated based on the min_area parameter, and only contours exceeding this threshold are retained and drawn on the final mask. This allows for the exclusion of artifacts while preserving fragmented wounds (multiple ROIs).

cnts, _ = cv2.findContours(mask, cv2.RETR_EXTERNAL, cv2.CHAIN_APPROX_SIMPLE)
final = np.zeros_like(mask)
area_threshold = (mask.shape[0] * mask.shape[1]) * (params.get(“min_area”, 10) / 10000.0)
if cnts:
    valid = [c for c in cnts if cv2.contourArea(c) > area_threshold]
    if valid:
       cv2.drawContours(final, valid, -1, 255, -1)
return final

#### Preprocessing: wounds alignment

Aligning wounds to the center of the image reduces errors when comparing different time points and, consequently, in velocity calculations. The alignment function is defined in the align_data method, which relies on NumPy matrix operations. The algorithm assumes vertical wounds; based on this assumption, it centers the horizontal coordinate of the gap’s centroid. The image is binarized (0 and 1), and the sum of white pixels (1, open area) is calculated along the y -axis for every x position. This produces a vector representing the topological profile of the scratch. Once the maximum (or the center of the maximum plateau) w_center is identified, the necessary shift is calculated as in Equation 2:

$$shift=\frac{Width}{2}-w_{center}$$ (2)

Using this value, a matrix circular shift operation is performed, moving the final band of width “shift” to the beginning of the matrix.

def align_data(self, img_bgr, mask):
    mask_n = mask.astype(np.float32) / 255.0
    sums = np.sum(mask_n, axis=0)
    if np.max(sums) == 0: return img_bgr, mask
    w_center = int(np.round(np.mean(np.where(sums == np.max(sums))[0])))
    shift = int(np.round((mask.shape[1] + 1) / 2)) - w_center
    return np.roll(img_bgr, shift, axis=1), np.roll(mask, shift, axis=1)

It is important to note that both the alignment and the subsequent velocity calculation strictly assume a vertical orientation of the wound. It is the user’s responsibility to pre-rotate any images exhibiting significant deviations from the vertical axis prior to importing them into WoundPy.

#### Calculation: absolute velocity

The software calculates the mean cell front advancement velocity based on free area variation. Unlike simple Closure Rate (often expressed in %/h), WoundPy converts this data into real physical velocity ( μm/h or mm/h ) assuming a bilateral closure model. The methods for calculating absolute velocities are defined as _compute_initial_final_vectors and _compute_over_time_vectors, both leveraging NumPy for matrix operations.

In the first case, for each pair of time points ( $T_{start}$ and $T_{end}$ ), the algorithm retrieves the respective binary masks, $M_{start}$ and $M_{end}$ . A “differential mask” ( Diff ) is computed to identify the pixels of the newly covered area (i.e., pixels where the wound has “healed”). The logic operation is described in Equation 3:

$$Pixel_{healed}=\left(Pixel_{M_{start}}==\text{Wound}\right)\wedge \left(Pixel_{M_{end}}==\text{Cells}\right)$$ (3)

In the code, the operation performed is diff = m0 - (2 * mf), resulting in a matrix with a value of 0 at pixel positions covering the area at $T_{start}$ , one on the newly covered area, and −1 on the area still open at $T_{end}$ .

def _compute_over_time_vectors(self, buf):
    df = self._buf_to_df(buf)
    px_per_um = self._get_px_per_um()
    for (rep, cond), group in df.groupby([“Replica”, “Condition”]):
        times = sorted(group[“Time_Num”].unique())
        if len(times) < 2: continue
        idx0 = group.loc[group[“Time_Num”] == times[0], “idx”].values[0]
        ref_shape = buf[idx0][“Mask”].shape
        for i in range(len(times) - 1):
           t_curr, t_next = times[i], times[i + 1]
           idx_c = group.loc[group[“Time_Num”] == t_curr, “idx”].values[0]
           idx_n = group.loc[group[“Time_Num”] == t_next, “idx”].values[0]
           mc = buf[idx_c][“Mask”].astype(np.float32) / 255.0
           mn = buf[idx_n][“Mask”].astype(np.float32) / 255.0
           mc = self._ensure_same_size(ref_shape, mc)
           mn = self._ensure_same_size(ref_shape, mn)
           diff = mc - (2 * mn)
           gray_counts = np.sum(diff == 1, axis=1)
           delta_t = max(t_next - t_curr, 1.0)
           vel_vec = gray_counts / (px_per_um * 2 * delta_t)
           self.velocity_over_time_data.append({“Replica”: rep, “Condition”: cond,
             “T_Start”: t_curr, “T_End”: t_next, “Vector”: vel_vec})

Subsequently, rather than summing the total area, the algorithm sums the healed pixels for each single horizontal row (for each y value) of the image. This generates a count vector, gray_counts, where each element represents how many pixels were “closed” at that specific wound height. This approach makes the calculation robust against irregular wound topology and independent of horizontal alignment, provided the wound at $T_{end}$ is completely contained within the wound at $T_{start}$ . In this regard, alignment minimizes errors associated with lateral shifts.

For each row y , the front velocity $v\left(y\right)$ is calculated assuming that the reduction in wound width results from the simultaneous advancement of two opposing fronts (left and right). The formula applied is described in Equation 4:

$$v\left(y\right)=\frac{gray\_counts\left(y\right)\cdot S_{factor}}{2\cdot \Delta t}$$ (4)

Where $gray\_counts\left(y\right)$ is the number of pixels covered by cells on row y in the time interval $\Delta t=T_{end}-T_{start}$ , $S_{factor}$ is the user-defined physical scale factor, and the division by two accounts for the two migration fronts.

Since each image row provides an independent velocity value, an image with a height of 1000 pixels yields a spatial sample of 1000 velocities, enabling the construction of histograms and Gaussian curves that map the distribution of migration velocities along the wound edge. The mean velocity for each image is considered the mean absolute front velocity for that replicate. Importantly, the associated standard deviation (SD) reflects the spatial and topological heterogeneity of the migration front along the y-axis, rather than biological or statistical confidence.

In the second method, the algorithm iterates through all consecutive available time points ( T0 to T1 , T1 to T2 , …). For each interval i , it calculates a local velocity vector $v_{i}$ , and the mean of this vector is saved as a scalar value. This allows for the distinction, for example, between constant migration and migration that is slow in the early hours of treatment and accelerates subsequently.

def _compute_initial_final_vectors(self, buf):
   df = self._buf_to_df(buf)
   px_per_um = self._get_px_per_um()
   for (rep, cond), group in df.groupby([“Replica”, “Condition”]):
      t_min, t_max = group[“Time_Num”].min(), group[“Time_Num”].max()
      if t_min == t_max: continue
      idx0 = group.loc[group[“Time_Num”] == t_min, “idx”].values[0]
      idxf = group.loc[group[“Time_Num”] == t_max, “idx”].values[0]
      m0 = buf[idx0][“Mask”].astype(np.float32) / 255.0
      mf = buf[idxf][“Mask”].astype(np.float32) / 255.0
      mf = self._ensure_same_size(m0.shape, mf)
      diff = m0 - (2 * mf)
      gray_counts = np.sum(diff == 1, axis=1)
      delta_t = max(t_max - t_min, 1.0)
      self.velocity_vectors_dict[f”R{rep}_{cond}”] = gray_counts / (px_per_um * 2 *
      delta_t)

#### Statistical analysis and PDF generation

To characterize the heterogeneity of cell migration within the population, WoundPy aggregates the row-wise absolute velocity vectors derived from the Initial-Final analysis in order to present such variability in empirical probability density functions. The method _plot_velocity_pdf performs a data pooling operation where, for each experimental condition, velocity values from all biological replicates are combined into a single statistical dataset all_values ( $N_{total}\approx {\text{Image}}_{\text{Height}}\times N_{replicates}$ ). While this pooling effectively visualizes the overall morphological and migratory variability across the entire cell population, it is important to clarify that any cross-condition statistical inference must be based on replicate-level summaries. For formal statistical testing between treatments, the true biological replicate serves as the independent experimental unit (e.g., n = 3 or 4), avoiding pseudoreplication. The empirical probability distribution of migration velocities is computed using the NumPy library via histogram analysis (np.histogram) with a resolution of 100 bins. The histogram is normalized (density = True) to ensure that the integral of the function equals 1, defining it as a Probability Density Function (PDF). For statistical modeling, the tool leverages the scipy.stats module from the SciPy library (Virtanen et al., 2020). Specifically, the norm.fit function is employed to estimate the population parameters - mean μ and standard deviation σ - by minimizing the likelihood function on the empirical data. When the “PDF + fitted” option is selected, these parameters are passed to the norm.pdf function to generate and overlay the theoretical Gaussian distribution curve $N\left(\mu ,\sigma^{2}\;\right)$ onto the empirical plot, allowing for a visual assessment of the normality of the migration behavior.

def _plot_velocity_pdf(self, out_dir, fit_gaussian=False):
    try:
        [...]
        all_values = []
        for vec in self.velocity_vectors_dict.values():
            all_values.extend(vec)
        [...]
        for i, cond in enumerate(conditions):
            vectors = []
            replicas = self.setup_data.get(“replicas”, [])
            for r in range(len(replicas)):
               key = f”R{r + 1}_{cond}”
               if key in self.velocity_vectors_dict:
                   vectors.append(self.velocity_vectors_dict[key])
          if not vectors: continue
          data = np.concatenate(vectors)
          counts, bins = np.histogram(data, bins=100, density=True)
          mu, std = norm.fit(data)
          plot_items.append({[...] “counts”: counts, “mu”: mu, “std”: std})
          [...]
          if fit_gaussian:
            pdf_fitted = norm.pdf(x_range, item[“mu”], item[“std”])
    [...]

## Results

### Tool structure

Upon launching WoundPy, a Welcome Page is displayed.

Clicking “Start New Analysis” opens the Experimental Setup Page, where users can define experimental time points (hours) and conditions, as well as specify the directories containing the WHA images. For experiments with numerous time points, the “Auto (discrete)” function generates equally spaced time points based on the total experiment duration and the desired number of points (e.g., a 72-h experiment with five points results in 0 h, 18 h, 36 h, 54 h, and 72 h). Condition names must exactly match the substrings within the image filenames or folder names.

The software supports three distinct directory structures (Modes) for individual replicate folders. For the “Flat (all in one)” mode the software expects all images to be contained within a single root folder. The “Nested by Condition” mode is particularly useful for automated acquisition setups with numerous time points, where organization by condition is logical. Conversely, the “Nested by Timepoint” mode facilitates manual acquisition workflows, where images for all conditions are typically captured sequentially at each specific time point. Accepted image extensions are.png, .jpg, .jpeg, .tif, .tiff and.bmp. The expected directory structures for these modes are schematized in Supplementary Figure S1A.

Proceeding to the ROI and Calibration Page, the user is prompted to set the conversion factor in pixels/mm or pixels/μm (the default value is one pixel). Clicking “Detect and Review ROIs” opens a dialog window where each image can be selected to interactively adjust ROI detection parameters. These parameters, adjustable via sliders, include variance radius, variance threshold, contrast, and minimum area. Specifically, the Variance Radius defines the neighborhood size for texture calculation: increasing this value smooths the detection map, reducing noise sensitivity but potentially blurring the precise wound edge. The Variance Threshold sets the sensitivity for distinguishing cellular texture from the background; increasing this value treats lower-texture regions as empty space (expanding the detected wound area), while decreasing it captures fainter cellular structures. Contrast enhancement improves feature visibility before processing, aiding in the detection of cells with low refractive indices. Finally, the Minimum Area parameter acts as a size filter; increasing the value of this parameter eliminates isolated artifacts or debris within the gap. Since image characteristics vary depending on microscope quality, magnification, and the refractive properties of the culture medium (influenced by volume and pH), different images may require specific parameter tuning. WoundPy enables dynamic adjustment of these values, granting the operator full control over ROI definition correctness. Additionally, complete wound closure can be manually indicated by forcing the area value to 0. The dynamic ROI tuning window is shown in Supplementary Figure S1B.

Upon saving, the workflow proceeds to the Analysis Output Page, where the user selects the desired outputs. First, the wound alignment preprocessing option can be toggled: this operation aligns wounds from the same replicate and condition across different time points, mitigating human error in centering the field of view during light-microscopy acquisition. The possible outputs are described below; in all cases, a.txt log file recording the ROI definition parameters is saved in the output directory, thus making the analysis fully replicable. The Analysis Output Page is shown in Supplementary Figure S1C.

### Outputs

#### RAW data (.csv)

These outputs provide.csv files containing absolute areas, normalized areas with respect to T0 (set to 100%), mean width values, mean front velocities between the initial and final time points, and interval velocities (if more than two time points are present). Where applicable, means and standard deviations are included.

#### Plots

**FIGURE 2:**
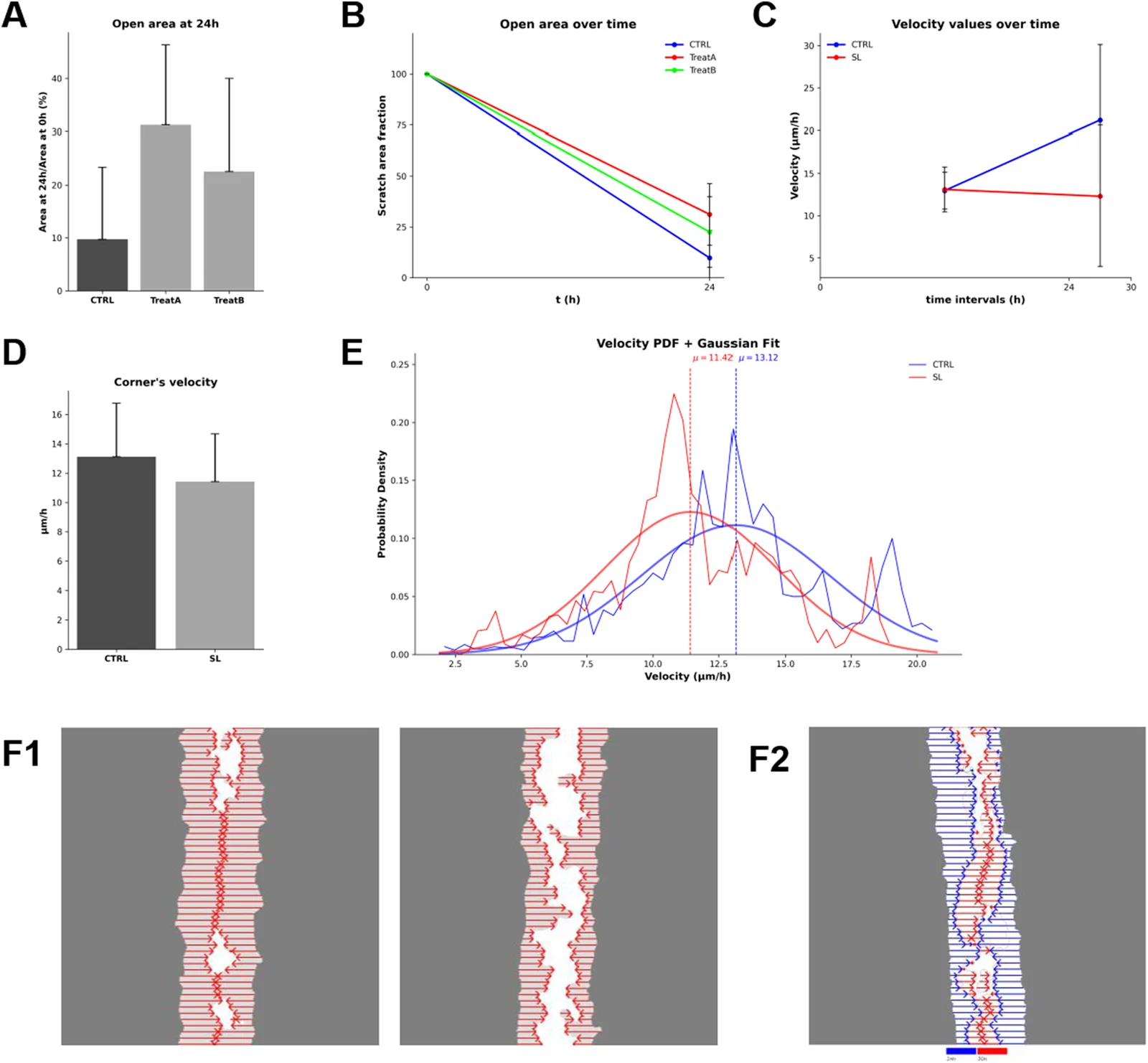
The available plots are shown, taking Setup 1 and 2 as examples. **(A)** “open area at final timepoint” histogram, **(B)** “open area over time” plot, **(C)** “velocity values over time” plot, **(D)** “corner’s mean velocity” histogram, **(E)** Probability Density Function plot for all absolute velocities of a specific condition, pooling all replicates; in the “PDF + fitted”“ option, the Gaussian curve is also displayed, as shown in figure. **(F)** Vector maps represent wound closure more effectively: in F1 the CTRL (left) and treated (right) conditions show efficacy in inhibiting cell motility by the treatment; in F2 the over time closure of the CTRL condition can be visualized at different times with the “Vectors over time” output.

These outputs generate time-course graphs and histograms for percentage areas and absolute velocities, as well as Probability Density Functions (PDFs) with relative mean values for all replicates (see Figures 2A–E). Time-course plots display the mean values of replicates at different time points for each condition, while histograms present data on percentage area reduction at the final time point and mean velocity between the initial and final points with standard deviations, where applicable. PDF plots represent the abundance distributions of absolute mean velocity values (initial-final), separated by condition, considering all absolute values along the wound fronts of all replicates. The “Fixed” output option allows for the overlay of Gaussian fits sharing the same mean and standard deviation.

#### Binary masks, vector maps and annotated images

The Binary Masks outputs provide the binary masks resulting from ROI detection, as well as their (eventual) post-alignment merged versions (see Supplementary Figure S2A).

The Vector Maps outputs offer an alternative, aesthetic visualization of the binary masks: similar to the masks described above, binary masks are overlaid with vectors indicating the direction of wound closure. This facilitates an immediate visual comparison between different conditions (see Figure 2 F).

The Annotated Images outputs provide images with wound contours overlayed as defined during ROI detection, along with annotations of the mean vertical wound fronts and their respective widths on each image (see Supplementary Figure S2B).

#### Scratch topography

**FIGURE 3:**
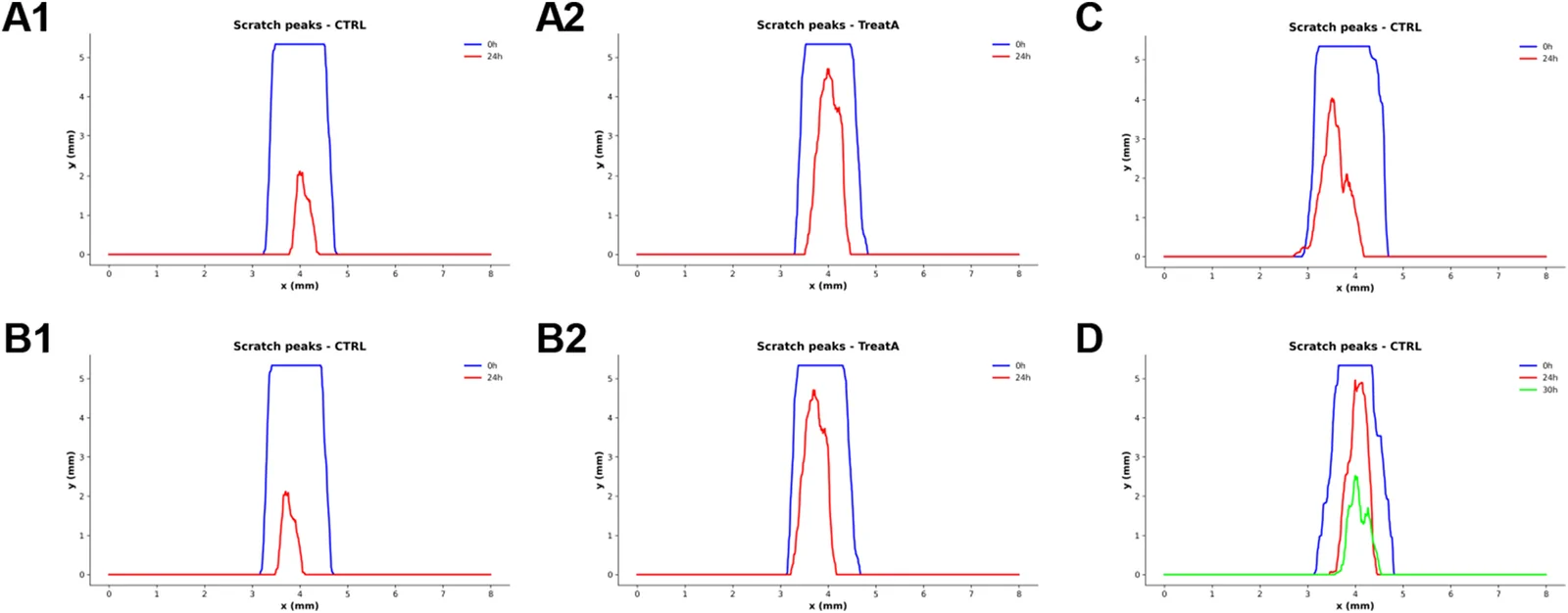
Scratch topography offers the possibility to evaluate different aspects. **(A)** Scratch peaks aligned for CTRL (A1) and treated (A2) conditions clearly show a greater reduction in open area in the treated one. **(B)** The same peaks before the alignment preprocessing demonstrate a possible human error in plate physical alignment during optical microscope acquisition. **(C)** It should be noted how, in the case of this not-aligned condition, the overlapping of profiles on the left would have led to an error in velocity estimation after calculating the newly covered areas. **(D)** Peaks decreasing over time show continuous closure up to 30 h.

This innovative generated plots display, for each x -axis value, the total linear measure of open area at that coordinate (see Figure 3). Each plot thus features a plateau at time T0 centered in the image, while for subsequent times, these plateaus narrow into peaks as the wound closes. If alignment is selected, all peaks will be centered; otherwise, each will be located at a different abscissa depending on the shift present during image acquisition. Since peak width and height correspond to the open area, these topological plots represent an additional data presentation modality.

#### ImageJ comparison

**FIGURE 4:**
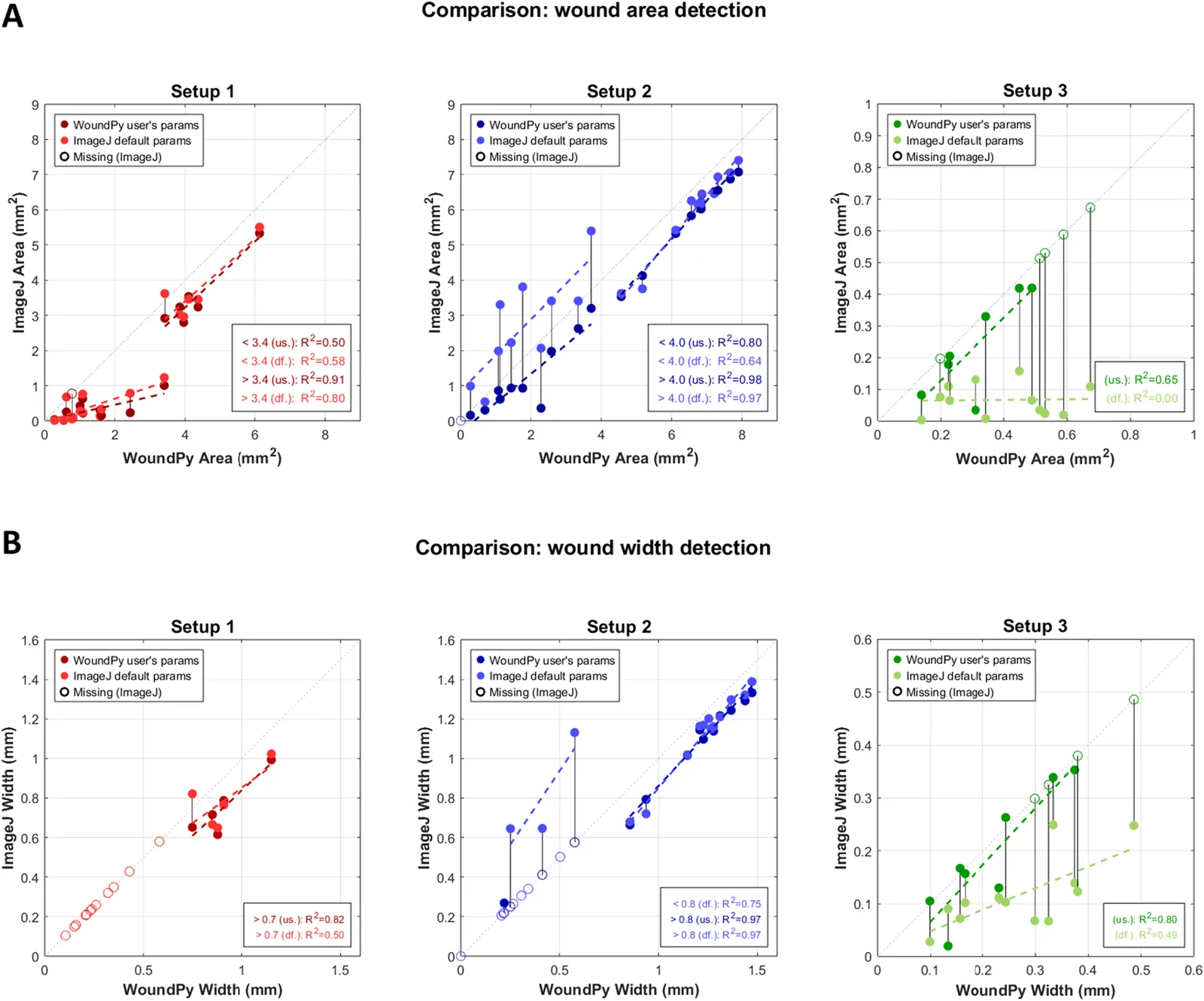
**(A)** Each point represents different wound area measurements on the same image. The x -coordinate represents the measurement obtained via WoundPy using user-defined parameter settings, while the y -coordinate is the measurement obtained with ImageJ in the following two modes: using the same parameters selected for that image in WoundPy (dark circles) and using the default parameters of the ImageJ Wound Healing Size tool (light circles). Empty circles represent area measurements performed with WoundPy that lack the corresponding ImageJ data due to the tool’s inability to detect the ROI. Points below the quadrant bisector represent an underestimation by ImageJ, whereas points above it represent an overestimation. Linear fits for two distinct populations across the two ImageJ methods were calculated (with reported $R^{2}$ ) for each setup. **(B)** Each point represents different wound width measurements on the same image. The coordinates have the analogue meaning as (A). Empty circles represent width measurements performed with WoundPy that lack the corresponding ImageJ data due to the tool’s difficulty in handling fragmented ROIs. Linear fits for two distinct populations across the two ImageJ methods were calculated with reported $R^{2}$ for setup 2, whereas for setup 1, data were insufficient for fitting the population at timepoints T>0h .

Images from three different experimental setups (see Methods) were analyzed using both WoundPy and the Wound Healing Size tool of ImageJ (Suarez-Arnedo et al., 2020) (hereinafter referred to as ImageJ) to provide a comparison of their estimated area and width measurements. Rather than claiming an absolute ground truth, we established a reference baseline representing expert-supervised segmentation. WoundPy measurements were performed using this expert-supervised semi-automatic approach. Since human validation will always remain the final step following the application of any analytical tool, in the case of WoundPy, this step is directly integrated into the dynamic parameter definition phase. To validate the robustness of the manual tuning, we assessed inter-operator variability by having three researchers independently perform the dynamic parameter definition on a representative subset of images (the first three replicates of Setup 2). This analysis demonstrated a high inter-operator agreement, yielding a mean Pearson’s *r* of 0.972 and a mean Coefficient of Variation (CV) of 13.69%. The raw data files used for this correlation assessment are provided in the “Inter-operator variance” folder, alongside the main datasets. Then, for each image, segmentation parameters (radius, threshold, contrast) were dynamically adjusted by an experienced researcher until the detected ROI boundaries visually coincided perfectly with the biological wound edges. Since WoundPy stems from the need to more rapidly identify the optimal parameters for use in ImageJ, the exact parameters determined via WoundPy were subsequently input into the ImageJ tool to ensure a direct comparison under identical settings. This approach specifically isolates and highlights the algorithmic differences between the two software tools, evaluating differences relative to the expert-supervised WoundPy segmentation rather than evaluating absolute accuracy against an independent standard. ImageJ measurements were therefore performed first by applying the same parameters selected for WoundPy, and subsequently using the default parameters of the ImageJ tool (radius = 20, threshold = 100, percentage of saturated pixels = 0.001). A value of contrast = 1 in WoundPy corresponds to an ImageJ value of percentage of saturated pixels = 0.001. For the third dataset, it was necessary to apply a threshold value in ImageJ that was 10 times higher than the one determined using WoundPy; indeed, given the specific image quality of this dataset, the original value failed to yield a proper definition of the Region of Interest (ROI). In instances where the ROI was fragmented, ImageJ analysis was restricted to the largest detected area: in such cases, ROI fragments were manually merged to measure the total area, though the width measurement remained unavailable; this primarily affected images during the healing phase ( T>0h ). Furthermore, the area measurement was also occasionally unavailable (Setup 3) due to ImageJ’s inability to detect the ROI with either default or user-defined parameters. Figure 4 A presents the graphical results of the area comparison, while Figure 4 B displays the width data.

**FIGURE 5:**
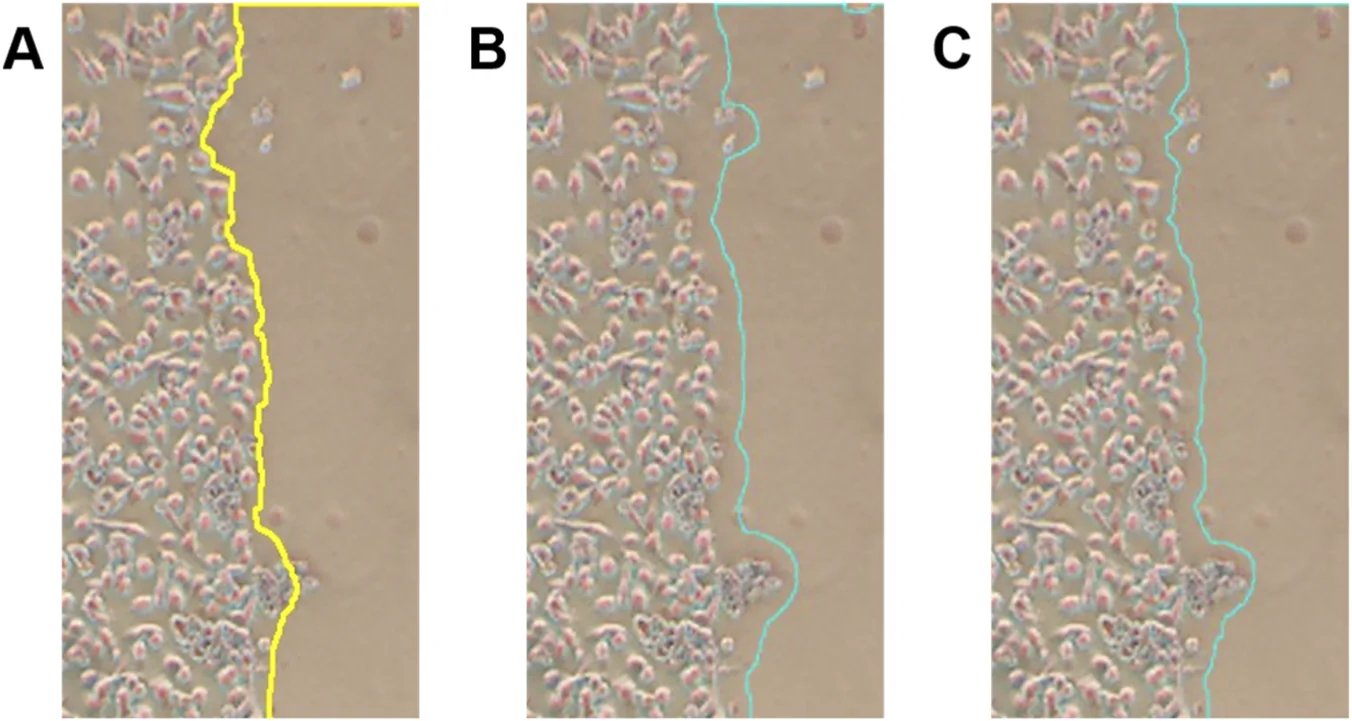
The three ROIs compared are detected on the same image using WoundPy **(A)** and ImageJ with two different methods: user-defined parameters **(B)** and default settings **(C)**. ImageJ’s systematic underestimation during the operation is visualized.

The area analysis revealed different behaviors of ImageJ compared to WoundPy depending on the timepoint of the analyzed image: for images at T0 ( $x\;>\;3.4\;mm^{2}$ in setup one and $x\;>\;4.0\;mm^{2}$ in setup 2), ImageJ seems to performs a measurement similar to WoundPy with a slight underestimation; for images at subsequent timepoints, the measurement error by ImageJ increases as the size of the fragmented wound increases. To determine if the ImageJ error is significant compared to the WoundPy measurement, linear regressions of the different subpopulations were evaluated, yielding the equations reported in Table 2A. Statistical tests were performed on the intercept and slope of the simple linear regression using one-sample Student’s t-tests. The intercept q test against a null hypothesis of q=0 verifies the presence of a constant bias, i.e., a systematic error between the two methods, while the slope m test against the null hypothesis of m=1 determines the existence of a proportional bias, i.e., an error that increases with varying ROI size. Statistical analysis shows that ImageJ underestimates areas when the ROI is small and fragmented in setup 1, and this error grows as the total ROI area increases. Conversely, underestimation is systematic for T0 images in setup 2 as also shown in Figure 5; as the ROI area increases, the use of default parameters in this case decreases the error ( m>1 , values approach the bisector). Underestimation or overestimation in other cases is not significant.

**TABLE 2:** Reports the comparison data for the different tools.

A	Setup	Population	IJ params	Linear fit	$R^{2}$	Slope vs. 1	q Vs. 0
​	1	$x<3.4\;mm^{2}$	User defined	y=0.24x−0.02	0.512	***	ns
​	1	$x<3.4\;mm^{2}$	Default	y=0.34x−0.04	0.585	***	ns
​	1	$x>3.4\;mm^{2}$	User defined	y=0.93x−0.51	0.908	ns	ns
​	1	$x>3.4\;mm^{2}$	Default	y=0.88x−0.12	0.800	ns	ns
​	2	$x<4.0\;mm^{2}$	User defined	y=0.77x−0.18	0.833	ns	ns
​	2	$x<4.0\;mm^{2}$	Default	y=1.17x+0.48	0.757	ns	ns
​	2	$x>4.0\;mm^{2}$	User defined	y=1.10x−1.41	0.983	ns	***
​	2	$x>4.0\;mm^{2}$	Default	y=1.21x−2.04	0.967	*	**
​	3	-	User defined	y=0.99x−0.07	0.654	ns (1:1)	ns
​	3	-	Default	y=0.01x−0.06	0.001	***	ns

B	Setup	Population	IJ params	Linear fit	$R^{2}$	Slope vs. 1	q vs. 0
​	1	x<0.7 mm	User defined	Insufficient data	-	-	-
​	1	x<0.7 mm	Default	Insufficient data	-	-	-
​	1	x>0.7 mm	User defined	y=0.91x−0.07	0.824	ns	ns
​	1	x>0.7 mm	Default	y=0.71x−0.14	0.496	ns	ns
​	2	x<0.8 mm	User defined	y=1.23x−0.00	1.000	p≪0.001	p≪0.001
​	2	x<0.8 mm	Default	y=1.86x+0.02	0.950	*	ns
​	2	x>0.8 mm	User defined	y=1.07x−0.21	0.968	ns	*
​	2	x>0.8 mm	Default	y=1.18x−0.33	0.970	*	**
​	3	-	User defined	y=1.07x−0.04	0.805	ns (1:1)	ns
​	3	-	Default	y=0.41x+0.01	0.491	***	ns

Table 2: **(A)** Linear fit values of area measurements are reported for the different populations and analysis methods for both experimental setups. Significance test results are also reported: in setup 1, analyses performed with both methods on the small area population carry an error that increases linearly and significantly compared to WoundPy; in setup 2, analyses performed with both methods suffer from significant error on the T0 population due to systematic underestimation; in Setup 3, the detection performed using default parameters fails regardless of the actual size of the area. **(B)** Linear fit values of width measurements are reported for the different populations and analysis methods for both experimental setups. Significance test results are also reported. In setup 1, analyses performed with both ImageJ methods on the small width population yielded no data. In setup 2, the error is significant for the small width population analyzed with default parameters, while analyses performed with both methods suffer from significant error on the T0 population due to systematic underestimation. In Setup 3, the underestimation of the width when using default parameters is systematic. Significance levels are reported in the table as ns: p>0.05 , *p<0.05 , **p<0.01 , ***p<0.001 . When p≪0.001 , the comparison resulted in a virtually null p-value due to the zero-inflated nature of the distribution in the healed condition.

ImageJ measurements that failed to detect the wound width were excluded from the regression analysis to prevent distortion of the linear fit, but for completeness were retained in the graphical representation as the WoundPy measure only. The width analysis also revealed differences in trends across the different subpopulations at T0 and T>0h (threshold at x=0.7 mm for setup one and x=0.8 mm for setup 2). Table 2B reports the corresponding linear regressions. Statistical analysis indicates that underestimation is systematic in setup two for T0 images, while the error is proportional with overestimation for T>0h images analyzed with default parameters. As with the area, in the case of width, the underestimation error decreases as the T0 wound grows ( m>1 ) when the analysis is performed with default parameters.

**FIGURE 6:**
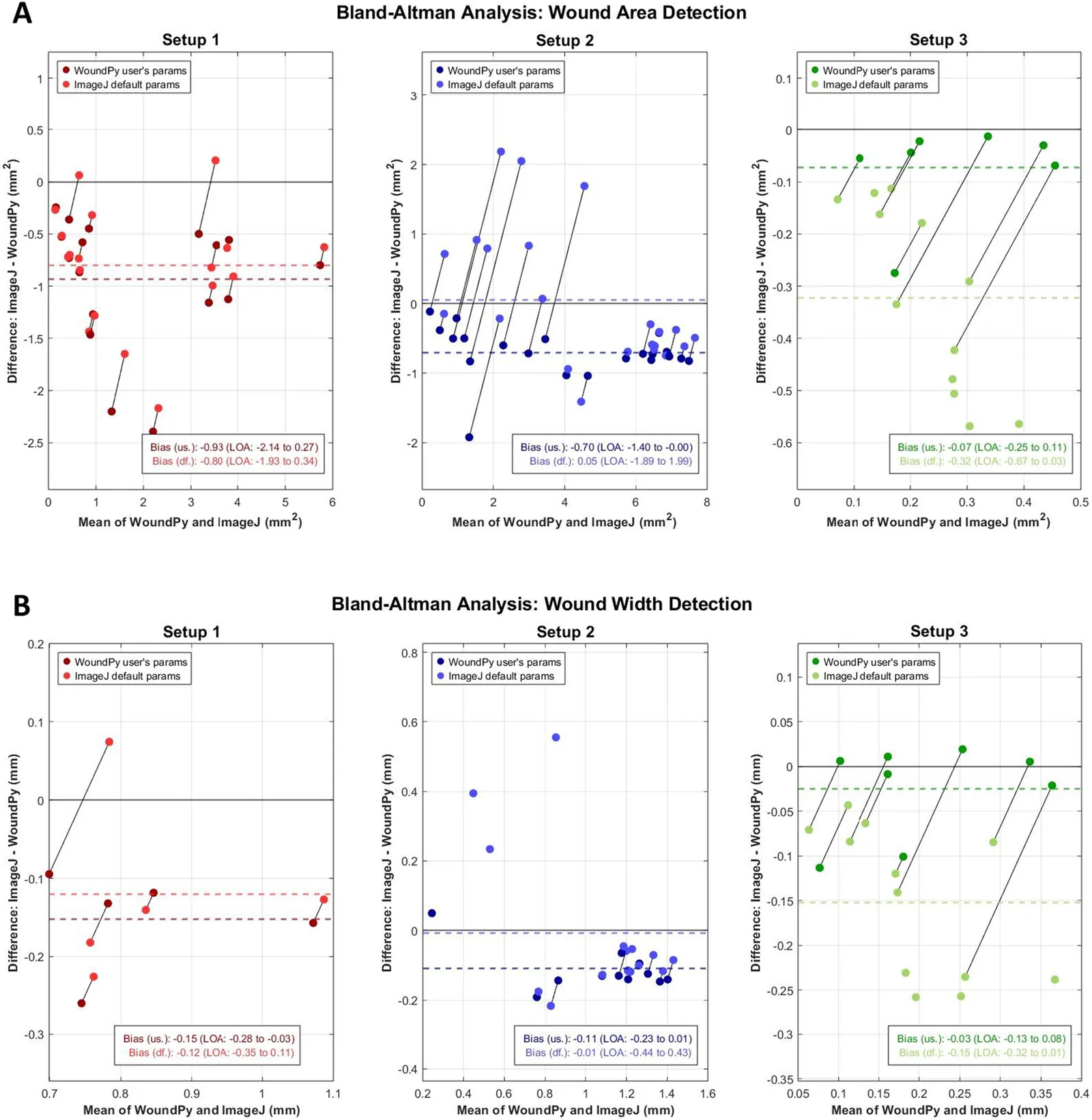
Bland-Altman plots for wound area **(A)** and width **(B)** comparison. The plots assess the agreement between measurements obtained with WoundPy and ImageJ across the three experimental setups. The x-axis represents the mean of the measurements from the two tools, while the y-axis shows the difference (ImageJ - WoundPy). Dark points represent ImageJ analysis using WoundPy user-defined parameters, while light points represent ImageJ default parameters. Solid lines connect measurements from the same image. The solid black horizontal line at zero indicates perfect agreement. Dashed colored lines represent the mean difference (bias) for each method, with negative values indicating an underestimation by ImageJ compared to WoundPy. The mean bias and 95% Limits of Agreement (LOA) are reported in the corresponding boxes for each condition.

To further evaluate the agreement between WoundPy and the standard ImageJ tool, a Bland-Altman analysis was performed on both wound area and width measurements (see Figure 6). The Bland-Altman plots visually confirm the systematic negative bias (underestimation) introduced by ImageJ relative to WoundPy across the different experimental setups. The solid black line represents perfect agreement (zero difference), while the dashed lines indicate the mean bias for each parameter setting. The predominantly negative bias values, coupled with wide Limits of Agreement (LOA), clearly illustrate that ImageJ consistently underestimates the wound size. Furthermore, in several cases (such as in Setup one and Setup two for the area), the negative difference visibly increases as the mean wound size grows, visually corroborating the proportional bias identified by the linear regression analysis.

## Discussion

Despite current analysis software tools being frequently labor-intensive and inaccurate, the Wound Healing Assay (WHA) remains an indispensable cornerstone of basic research, as it allows for the examination of complex biological processes aimed at a fast identification of compounds capable of inhibiting cell migration. The WHA has often been criticized as obsolete due to low reproducibility associated with operator errors and automated tools for Region of Interest (ROI) detection (Vang Mouritzen and Jenssen, 2018; Guy et al., 2017). However, the optimization of this technique from an analytical perspective constitutes a value of fundamental importance in cell migration research, given the assay’s inherent simplicity of execution.

With the development of WoundPy, we provide a comprehensive workflow that fully automates both the analytical calculation and data plotting processes. Unlike other software solutions that often require complex scripts or fragmented plugins, WoundPy is distributed as a standalone executable capable of managing datasets efficiently. The robustness of the tool was successfully validated on three real biological datasets, encompassing diverse experimental conditions and biological replicates.

A distinct technical strength that sets WoundPy apart from basic analysis tools is the implementation of a preprocessing phase dedicated to wound alignment. While software facilitating temporal overlay analysis often necessitates expensive automated microscopes to guarantee a fixed field of view, WoundPy democratizes this capability by computationally correcting the human error intrinsic to manual acquisition with standard optical microscopes. This function is crucial for data quality: without alignment, plate positioning errors can generate overlapping topological profiles that falsify the calculation of newly covered areas and, consequently, the estimation of migration velocity. This innovation renders the analysis reliable even in laboratories equipped with standard instrumentation.

WoundPy marks a significant advancement over classical methods that are limited to reporting the percentage Closure Rate or mean front velocity (often manually drawn), metrics that offer only relative measures or are subject to operator error. Our approach converts the variation in cell-free area into an absolute physical velocity ( μm/h or mm/h ) by analyzing wound closure row-by-row along the image height. This method generates a broad statistical sample of velocity vectors for each replicate, allowing for the derivation of real physical data that is directly comparable across different experiments.

The superiority of the expert-supervised semi-automatic segmentation implemented in WoundPy emerged clearly in the direct comparison with the standard ImageJ tool, as illustrated in the quantitative results (Figure 4). Our validation demonstrated that while ImageJ performs adequately on standardized samples (T0), it introduces systematic errors in complex images. Statistical analysis revealed that ImageJ, compared to WoundPy, tends to systematically underestimate wound area and width relative to the expert-supervised WoundPy segmentation when the gap becomes fragmented, with an error that grows proportionally to the complexity of the ROI, or when the initial contrast of the image is low. In contrast, WoundPy allows for dynamic and interactive parameter tuning for each individual image, ensuring that even the most irregular ROI fragments are correctly detected and included in the final calculation.

Furthermore, ImageJ forces the researcher to restart the entire pipeline even for a single small parameter change: WoundPy reduces analysis time by allowing real-time ROI modification. The generation of a log file guarantees substantial reproducibility of the analysis across all replicates, and their management through different folder nesting modes facilitates image handling in experiments with a large amount of data.

In spite of the significant advancements introduced by WoundPy, certain limitations inherent to the current algorithmic approach must be acknowledged. First, the preprocessing alignment module and the subsequent row-by-row absolute velocity calculation are mathematically optimized for vertically oriented wounds. While the software can handle slight deviations typical of manual scratching, significant deviations from the vertical axis may compromise the accuracy of the alignment and the resulting vector maps, necessitating that users pre-rotate such images before import. Secondly, the segmentation engine itself is built on variance-based texture analysis combined with human supervision, rather than Deep Learning (DL) models. While this keeps the tool lightweight and accessible - removing the need for high-end GPUs or massive training datasets - it implies that detection sensitivity depends on the local contrast between cells and the medium. Currently, WoundPy is distributed as a standalone Windows (.exe) and macOS (.app) executable to maximize accessibility for researchers without programming expertise, though this does limit native use on macOS or Linux.

Future developments of the tool will aim to potentially integrate pre-trained lightweight neural networks to enhance segmentation robustness in high-noise conditions.

## Data Availability

All image datasets, corresponding software outputs, ImageJ comparison data, the software source code and executable (.exe) files are available in an online repository on Zenodo (Galante et al., 2026).
