# Supplementary material for: WoundPy: an improved automatized method for in vitro cell motility investigation based on wound alignment and absolute velocity profiling
Source: Front Cell Dev Biol. 2026 Sep 7;14:1919916. doi: 10.3389/fcell.2026.1919916 (PMC13590394; doi:10.3389/fcell.2026.1919916)
Supplement: Supplementary file 1 [file Supplementaryfile1.docx]

**Supplementary**

Fig. S1: **(A)** Directory structures expected by the code based on the selected mode: a) Flat (all in one) mode; b) Nested by Condition mode; c) Nested by Timepoint mode. **(B)** Tuning window: the sliders on the right allow for real-time visualization of ROI identification based on the set values. **(C)** Analysis Output Page: each output is associated with an informative example that can be viewed by clicking the blue *i*-icon; a log window at the bottom provides information on the current analysis status.

Fig. S2: **(A)** Binary masks convey information about treatment efficacy using fewer images than the originals: the A1 and A2 images represent the binary merge respectively between CTRL and TreatA of a replicate from experimental setup 2 from T0 (black) to T1 (gray), showing a slowdown in closure for the treated condition; A3 and A4 represent the different closure stages respectively for conditions CTRL and SL of a replicate from experimental setup 1, passing through the different timepoints (T0 black, T1 dark gray, T2 light gray). **(B)** Requested the "Annotated images", WoundPy saves in the output directory (B1) the ROIs defined prior to analysis and (B2) the images overlaid with the mean front and its width.
